# Importance of Sock Type in the Development of Foot Lesions on Low-Difficulty, Short Hikes

**DOI:** 10.3390/ijerph16101871

**Published:** 2019-05-27

**Authors:** Ana Mª Pérez Pico, Ester Mingorance Álvarez, Rodrigo Martínez Quintana, Raquel Mayordomo Acevedo

**Affiliations:** 1 Department of Nursing, University of Extremadura, Plasencia, 10600 Cáceres, Spain; aperpic@unex.es; 2 Department of Anatomy, University of Extremadura, Plasencia, 10600 Cáceres, Spain; emingorance@unex.es; 3 Department of Statistics, University of Extremadura, Plasencia, 10600 Cáceres, Spain; rmartinez@unex.es

**Keywords:** sport injury, technical socks, temperature, hiking

## Abstract

*Background and objectives:* Foot lesions can be developed during hiking because of external factors. This makes it important to study the effect of hiking equipment on lesion development. *Materials and Methods:* Technical and non-technical socks were given to 109 hikers to wear during a short hike. Participants were examined at three stages of the hike to determine the development of dermal, muscle and nail lesions, temperature and perimeter in various areas of each foot. *Results:* The percentage of hikers without injuries was significantly higher among those wearing technical socks (*p*-value < 0.001). Differences were also observed in mean foot temperature, which was higher in participants wearing technical socks (*p*-value < 0.001). *Conclusion:* The results indicate that even on a low-difficulty, short-term sport activity, it is advisable to wear technical socks to prevent lesion development and keep the foot temperature more stable. Sock type was identified as an external conditioning factor in lesion development.

## 1. Introduction

Outdoor sports, especially activities in the natural environment such as hiking, mountaineering, and climbing, are very popular pursuits [1]. Participants are affected by many external factors during these types of activities. For example, outdoor hiking and indoor walking have different metabolic requirements [2]. The terrain of the hike and its relief also affect hikers [3]. Similarly, hikers are affected by variable weather conditions, including high doses of solar radiation [4]. Carrying a backpack, including factors such as backpack carriage design [5], weight [6], and load distribution [7,8], needs to be taken into account. Use of a stick while hiking [9], the choice and even the color of clothing [10] are further factors that can influence successful completion of a hike. Footwear type is another external factor that has been shown to affect sports activity [6,11].

These factors can be determinant in accidents and injuries of various levels of severity occurring among mountaineers [12,13]. One of the most frequent dermal lesions is the development of blisters in the feet due to sweat accumulation and friction during walking [14]. Applying antiperspirants to the feet to control sweating has been shown to be effective at reducing foot blister incidence in recruits during military training [15]. Lower limb muscle injuries [16] and injuries due to backpack use and weight are also frequent [6]. Development of nail lesions after long-distance walks has also been previously described [17].

In 2006, Zhong et al. reported that the study of textiles and their effect on the skin was still far from reaching solid conclusions. The authors highlighted the importance of determining the characteristics of the skin of each individual (surface roughness, hydration, adhesion between cell layers, etc.) [18]. New textiles that have been studied and developed since then may help to reduce the development of injuries [16,19] and improve the thermal comfort of footwear during sports activity [20]. Other authors have developed fabrics with antifungal and antibacterial properties [21]. Copper oxide nanoparticles have antibacterial activity [22] and bioceramic fibres are bacteriostatic [23]. The validity of a smart textile has been demonstrated for assessing pressure, plantar temperature, and joint angles in patients with diabetic peripheral neuropathy during habitual gait [24].

Hiking is highly recommended for improving fitness, especially in people with certain diabetic or vascular process [24,25,26]. However, health professionals in general, and podiatrists in particular, are treating more sports-related lesions, especially blisters, subungual hematomas, wounds, sprains, muscle problems, and chafing [27,28,29]. These lesions affect healthy hikers and could have serious consequences for at-risk patients [30].

Numerous studies have analyzed dermal lesions, particularly blister incidence, developed by military personnel wearing socks of different compositions [16,31,32] and by long-distance runners [33,34]. However, no field studies have been conducted to simultaneously analyze the prevalence of dermal, muscle, and nail lesions, or the effect on hikers of socks of various textiles during low-difficulty, short walks. Studies are needed to analyze the influence of external factors on the development of foot lesions and address ways to avoid these lesions.

It is highly important for health professionals to understand the factors involved in the development of foot pathologies during sports activity [16,28,35,36] and particularly during hiking. The main objective of this study was to analyze the dermal, muscle, and nail lesions that appeared and/or were developed in hikers’ feet on a low-difficulty, short walk with regard to the type of sock used (technical or non-technical).

## 2. Materials and Methods

### 2.1. Permission and Admission Criteria

All participants signed an informed consent form, and permission for the study was obtained from the University of Extremadura Bioethics Committee (reference 126/2016).

The inclusion criteria were: Being of legal age, not having current health problems that would hinder or prevent participation in the hike, and carrying a backpack weighing less than 3 kg. Participants were asked to use specific hiking footwear (light, flexible, soft-soled, with good grip, and breathable material) (low top hiking shoes), based on earlier studies [37,38,39]. They were also asked to do the entire hike following the instructions of an experienced guide and to not remove their footwear or socks before examination by a podiatrist.

### 2.2. Sock Type

Socks were of two types: Technical socks, designed for high performance sports use (Lurbel brand, models Tierra and Set), and non-technical socks for everyday use. The socks had different compositions: Tierra (50% regeneractiv^®^, 25% cool-teak, 17% polyamide ions, 8% lycra); Set (75% cotton, 17% polyamide, 8% lycra) and cotton (98% cotton, 2% elastane). The technical socks had reinforced weave in the toe, metatarsal, and heel areas.

### 2.3. Study Design, Route Description, and Participants

A double-blinded, randomized control trial was designed to analyze the influence of different types of socks (technical and non-technical) on the appearance of foot injuries after a low-difficulty, short-term sport activity. The study follows the CONSORT criteria. This work was included in the ClinicalTrials.gov databases with the ID: NCT03914274.

The researchers who did not intervene in the exploration process, registered the hikers previously and obtained data on age, sex, foot number, and health condition. They also randomly delivered technical or non-technical socks to each hiker. Before delivery, they removed from the socks all the labels with composition or references to blind the process to both hikers and explorers. Control hikers wore two non-technical socks while the rest of the hikers wore one type of technical sock on each foot. All the participants before the initial exploration had their socks put on for half an hour.

The hike was low-difficulty, over a short distance [40]. The first 14.5 km were on asphalt and the last 15.1 km on ground. The height range was 168 m and the pace was moderate, at 5 km/h. Cumulative elevation gain was 255 m and cumulative vertical descent was 147 m.

The sample comprised of 109 participants (53 men and 56 women). Two women wearing technical socks had to give up the hike for personal reasons. Anthropometric characteristics of the participants and the distribution of the socks, differentiating by sex and feet are shown in Table 1.

### 2.4. Study Variables

The qualitative variables analyzed were: Gender, body mass index (BMI) by rank, sock type, dermal lesions (all lesions developed during the hike, both keratopathies with keratinization disorder and dermatopathies without keratinization disorder), muscle lesions (all lesions developed during the hike), and nail lesions (all nail lesions developed during the hike, both traumatic and non-traumatic onychopathies). The height and weight data obtained were used to calculate BMI by rank, interpreted according to the World Health Organization classification [41] (Table 2).

The quantitative variables analyzed were: age, temperature of each foot measured in two aspects (plantar and dorsal), and perimeter of each foot, measured in four areas (dorsal aspect of toes, instep, heel, tibia).

### 2.5. Measuring Instruments

Participants were examined by trained podiatrists on three occasions to measure the study variables: At the start of the walk (km 0), at the end of the asphalt section (half way; km 14.5), and at the end (km 29.6). Data was kept in a form at all time (form included as Supplementary Data).

The height range and the pace hikers were controlled with a Garmin ETREX 20X GPS (Global Positioning System). The Perimeter was measured with a flexible, non-elastic tape measure (Lawton 18–0160, precision 1 mm). Weight was measured using scales (Tanita UM-076, precision 0.1 kg) and height was measured using the weight rod of different scales (SECA 704, precision 1 mm).

Temperatures were taken with an infrared thermometer (FTN Medisana, precision 0.18 °C), calculating the mean of the three measurements, with a mean error of the temperature of 0.1452.

### 2.6. Statistical Tests

The data were analyzed using IBM SPSS Statistics for Windows, Version 22.0 (IBM, Armonk, NY, USA). The statistical treatment consisted of data study and cleansing, and descriptive variable analysis. Normality test and Shapiro-Wilks test were performed with significant results (*p*-values < 0.044).

Non-parametric methods of longitudinal data in factorial experiment with nparLD Package in R have been used in previous studies [42] to check the interaction between sock and distance (km). In case of interaction depending on whether the samples were paired or independent, Friedman’s test and Kruskal-Wallis test was performed, respectively, both with Bonferroni correction for multiple testing. The effect size for the different variables was also calculated. First, for Kruskall-Wallis tests, the variation was between 0.443 and 0.888, and for Friedman tests, the variation was between 0.336 and 0.623. Therefore, in both cases, the effect size could be considered between median and high.

For the qualitative variables, the chi-square or Fisher’s exact test with a significance level of α = 0.05 was carried out. The variables analyzed by logistic regression were quantified using the ODDS Ratio (OR). From now on, *p*-values = 0.000 are less than 1 per 1000.

The maximum error in estimations calculated according to the sample size analyzed (109 individuals) is 0.093865 for the qualitative variables and 1.320571 for quantitative variables with a maximum standard deviation observed of 7.034.

## 3. Results

After completing the first part of the hike, the most frequent dermal lesions among participants were blisters and erosions. None of the other dermal lesions included in the evaluation (reddened skin, urticaria, crevice, and heloma) were detected. Significant differences were found between the percentage of dermal lesions in hikers wearing technical socks (6.6%) and non-technical socks (36.4%) (*p*-value = 0.000). The women had a higher percentage of dermal lesions (23.2%) than the men (7.5%) (*p*-value = 0.034), regardless of sock type. Moreover, the percentage of women with dermal lesions was significantly higher among those wearing non-technical socks (50%) than in those wearing technical socks (10.5% had lesion) (*p*-value = 0.002). Particularly, women presented more blisters in both right foot (RF) and left foot (LF) when they wore cotton socks compared to the Tierra and Set socks (RF *p*-value = 0.050, LF *p*-value = 0.042). In the case of men, there was no difference in any of the skin lesions at 14.5 km on any foot compared to the sock used (Table 3). The most frequent muscle lesions at the end of the first part of the hike were pain due to inadequate warm, inflammation, and muscle discomfort. No sprains or nail disorders were detected. No difference between muscle lesions analyzed and technical and non-technical socks were found. By sex, no differences were found regarding technical and non-technical socks or between technical socks (Table 3).

On the other hand, in the second part of the hike, a greater variety of dermal lesions was detected. The most prevalent were blisters, erosions, and reddened skin. Again, no reddened skin, urticaria, crevices, or helomas were detected with either technical sock. Differences between the percentage of dermal lesions among hikers wearing technical socks (21.1%) and non-technical socks (54.8%) were found (*p*-value = 0. 001). By gender, the percentage of women and men with dermal lesions was higher among those wearing non-technical socks (women 68.8% and men 40%) than among those wearing technical socks (women 26.3% and men 15.8%) but the differences only were significant in women (*p*-value = 0.006). Considering the sock used in each foot, women presented more reddened skin in both RF and LF if they used a cotton sock compared to the Tierra and Set (RF and LF *p*-values = 0.023). In the case of men, they presented more blisters in the LF if they used the Set sock rather than cotton or Tierra (*p*-value = 0.021) and more erosion in the RF if they used the cotton sock in comparison with the Tierra or Set (*p*-value = 0.018) (Table 4). A greater variety of total muscle lesions was also detected in the second part of the hike: Pain due to inadequate warm, inflammation, muscle discomfort, and sprain. No differences were found between any of the muscular injuries analyzed, and neither by sex for the technical and non-technical socks (Table 4).

Nail lesions, like onychocryptosis and subungual hematomas, were infrequent and developed only in the second part of the hike. No difference between any of the nail lesions analyzed and technical and non-technical socks was found. By sex, no differences were found (Table 4).

Regarding temperature, significant interactions were detected in males (*p*-values < 0.000) and indications of significance in females (*p*-values < 0.1). Moreover, the comparisons between sock types (*p*-value < 0.009) and distances (*p*-value < 0.005) showed significant differences, regardless of foot, measurement area and sex (Table 5 and Table 6, and Supplementary Data, Tables S1 and S2).

No significant differences were detected between technical socks models. However, significant differences were detected between each model of technical socks and the non-technical socks for all distances, areas of the feet, and sex. The greatest differences were detected at KM 0.

The differences in temperature were greater at the beginning of the hike than at the end. A significant increase in temperature from 0 km to 14.5 km was observed, and temperature stabilization occurred during the 14.5 km to 29.6 km section of the hike. In all types of socks, a significant increase was detected from the beginning to the end of the hike. However, magnitude of the increase in temperature, varied between the types of socks in all the foot areas measured; being superior in non-technical socks (about 8.5 °C) than in technical socks (about 1.2 °C) (Supplementary Data, Tables S1 and S2).

Notice, in the case of men, there was an increase in temperature in the plant of both feet from the middle to the end of the hike (LF *p*-value = 0.056, RF *p*-value = 0.091) although it cannot be considered statistically significant (Table 6).

The study of the perimeter of hikers’ feet analyzed in total and by gender, in relation to distance and sock type, did not reveal significant results (data not shown).

It was possible to study the relation between variables and predict the probability of lesions developing during the hike using logistic regression analysis (Table 7). Women had a higher probability than men of suffering skin lesions in hikes of up to 14.5 km. However, men hd a higher probability than women of suffering skin lesions on hikes of 14.5 to 29.6 km and muscle lesions on hikes of up to 14.5 km.

The non-technical socks group showed the highest probability of suffering dermal and muscle lesions, both from 0 to 14.5 km and from 14.5 km to the end of the hike. The probability of dermal and muscle lesions development in relation to the quantitative variables showed both direct and inverse relations. From 0 to 14.5 km, the BMI and age of hikers were directly and inversely related, respectively, both in dermal and in muscle lesions. In the second part of the hike, age continued to show an inverse relation with skin lesions, but no relation with BMI was identified (Table 7).

## 4. Discussion

Numerous studies have analyzed lesions and illnesses developed by hikers on long distance, endurance, and difficult walks. Boulware et al. studied the development of lesions and injuries after three days of mountain hiking [43], Choi et al. after 21 consecutive days of walking [17], Anderson et al. on long distance hikes [6], and Travis et al. on short walks on volcanic terrain [44]. However, no studies have addressed one-day or low-difficulty, short hikes, the type most recommended for improving physical health and reducing stress [45,46]. Data for the present study were collected by physical examination rather than through questionnaires filled in by hikers after completing a walk, as in some of the works identified [6,11,17,43,44,47].

The importance of both footwear and sock type has been demonstrated in comparative studies of socks made mostly from natural fibers (cotton or wool) or mostly from synthetic fibers (acrylic, polyester, and polypropylene) [16,32,33,34,47,48]. Socks with a higher synthetic fiber content (hydrophobic) show better results among runners wearing sports shoes [33,34], while socks with 50% wool (hygroscopic) perform better with military boots [32,47]. The present results show that technical socks cause fewer dermal lesions than non-technical socks, probably because hiking footwear breathes better than military boots and in a similar manner to sports shoes.

Numerous risk factors affect blister development, such as dampness, sweat, temperature, and friction [32,47]. Kirkham et al. showed that, in controlled conditions, increased surface skin hydration increases the rate of skin temperature change and the risk of blisters [14]. However, the present results show that technical socks caused fewer dermal lesions during a hike, even though they kept foot temperature higher than non-technical socks. This may be due to the lower temperature increase during the hike with technical socks and the greater ability of these socks to transport sweat away from the surface of the skin [33,49].

Despite the sample size and the distance of the hike, no differences were detected in hikers’ foot perimeters. In controlled conditions, McWhorter demonstrated an increase in foot volume in men after 10 min of treadmill walking [50]. The difference in the mean age of the two populations, and measuring perimeter rather than volume may have been determining factors.

Women had a higher percentage of foot lesions than men, despite the higher age and BMI of men. Differences due to sexual dimorphism between men and women are more determinant in the development of foot lesions than intrinsic factors such as age and BMI.

Both men and women had dermal lesions in the first 14.5 km, although prevalence was higher among women. These results are in agreement with those obtained by Patterson et al., Van Tiggelen et al., and Brennan et al. in military personnel [11,16,51]. Among those wearing of non-technical socks, the women also showed a greater temperature increase than the men, which could result in higher blister prevalence [14]. However, the limited temperature increase observed in women and men wearing technical socks, strengthens the idea that a lower temperature increase means fewer dermal lesions. Moreover, fabrics with synthetic fibers eliminate sweat better, reducing the prevalence of these lesions.

The percentage of total muscle lesions was higher in the first part of the hike than at the end. This may be due to insufficient warm up and stretching before starting. However, hikers’ muscles warmed up as they covered more distance, which may have helped to limit muscle lesions. Van Tiggelen et al. reported that a higher number of women suffered knee injuries after hiking [16]. These results are similar, because women in the present study had a higher percentage of muscle injuries than the men.

The low prevalence of nail lesions precluded significant differences. However, nail lesions were detected after participants had hiked 14.5 km. This shortens the distance of 580 km in which Choi et al. detected the loss of the nail plate as the only nail lesion [17].

The present study is unique in analyzing gender, sock type, BMI, and age of hikers as predictive factors of dermal and muscle lesions. Patterson reported that women have a higher ‘relative risk ratio’ than men for developing blisters, which was also found in the results obtained in the present study for dermal lesions in the first part of the hike [51]. However, men were more likely to have developed dermal lesions by the end of the hike. This could be due to physiological differences in men’s and women’s skin, given that men have thicker skin. Men also have less subcutaneous tissue than women [52]. This may have influenced the higher probability of men suffering muscle lesions at the start of the hike. With age, skin becomes thinner [53], but the present study established an inverse relation between age and the probability of suffering dermal and muscle lesions. Hiking experience gained over the years may play a decisive role, as well as participation in previous hikes [16]. Body mass index was also a direct predictive factor in foot lesions. Although no studies analyzing BMI as a predictive factor were identified, backpack weight has been determined as a risk factor in blister development [17]. Non-technical socks were revealed as a powerful predictive factor of dermal and muscle lesions in hikers. However, other studies reported that socks comprising mostly natural fibers reduce blister development [32,47].

In the light of the results obtained, it is essential to consider the type of activity when recommending a particular sock type. The differences determined by gender will help health professionals to individualize primary prevention and make recommendations based on the predictive factors identified in this study to reduce the prevalence of foot lesions.

## 5. Strengths and Limitations

There were certain strengths and limitations to our study. This was the first study that examined podiatric injuries developed in hikers according to the type of the socks used (technical and non-technical) during a hike at low difficulty and short distance. Our data is not based only on questionnaires, rather by evaluations of trained podiatrists who diagnosed all dermal, muscle, and nail lesions developed along the hike.

However, our findings should be interpreted with caution because the incidence of some injuries has been very low. Therefore, the logistic regression test was performed with grouped variables.

It was not possible to detect differences in foot perimeter, which probably requires longer and more difficult hikes or more sensitive measuring methods.

The research project that sponsored the completion of the hiking route allowed us to make a public call that was attended by the participants; therefore, sample size could not be adjusted or incremented. This question limits the conclusions reached in this study but opens the possibility of completing these data with further studies.

## 6. Practical Applications

The results show the importance of choosing an adequate type of sock according to the sex of the hiker and the distance of the route because that procedure would reduce the incidence of injuries during sport activity. Therefore, this study gives information to advise correctly in the election of an adequate sock for a specific sport.

## 7. Conclusions

Dermal, muscle and, nail lesions occur even on low-difficulty, short hikes and are more frequent in women than in men.

Technical socks are recommended for low-difficulty, short hikes because they reduce the development of foot lesions and keep foot temperature more stable.

Detecting differences in foot perimeter requires longer and more difficult hikes or more sensitive measuring methods.

Age, gender, BMI, and sock type are powerful predictive factors for the risk of suffering lesions. Health professionals can use these parameters to make valuable recommendations to patients to prevent and reduce the prevalence of foot lesions.

## Figures and Tables

**Table 1:** Anthropometric variables, total sock distribution and sock distribution by gender.

**Anthropometric Variables**		**Total**	**Male**	**Female**
Gender			48.6%	51.4%
Age (years)		44 ± 25	49 ± 21	36 ± 24
Height (meters)		1.7 ± 0.1	1.7 ± 0.1	1.6 ± 0.1
Weight (kg)		68.8 ± 19.4	81.0 ± 13.5	61.4 ± 7.8
BMI		24.9 ± 3.6	26.6 ± 3.7	23.5± 9.1
**Sock type distribution**		**Total %**	**Male %**	**Female %**
LF sock	Tierra	40.4 (*n* = 44)	35.8 (*n* = 19)	44.6 (*n* = 25)
	Set	29.4 (*n* = 32)	35.8 (*n* = 19)	23.3 (*n* = 13)
	Cotton	30.2 (*n* = 33)	28.4 (*n* = 15)	32.1 (*n* = 18)
RF sock	Tierra	29.4 (*n* = 32)	35.8 (*n* = 19)	23.3 (*n* = 13)
	Set	40.4 (*n* = 44)	35.8 (*n* = 19)	44.6 (*n* = 25)
	Cotton	30.2 (*n* = 33)	28.4 (*n* = 15)	32.1 (*n* = 18)

% = Percentage, ± = Standard deviation, kg = Kilograms, BMI = Body mass index, LF = Left foot, *n* = Sample size, RF = Right foot.

**Table 2:** Qualitative variables and categories.

Qualitative Variable	Category
Gender	Male
	Female
BMI	Underweight (<18.5)
	Normal weight (18.5–24.99)
	Overweight (25.0–29.9)
	Obese (>30)
Sock type	Technical (Tierra)
	Technical (Set)
	Non-technical (Cotton)
Skin lesion	Blister
	Erosion
	Reddened skin Urticaria
	Crevice
	Heloma
Muscle lesion	Pain due inadequate warm
	Inflammation
	Muscle discomfort
	Sprain
Nail lesion	Onychocryptosis
	Subungueal hematoma

**Table 3:** Prevalence of skin, muscle, and nail lesions during the 0 to 14.5 km of the hike by sex.

(0–14.5 km)		**Female**						**Male**					
		Tierra %		Set %		Cotton %		Tierra %		Set %		Cotton %	
**Skin Lesion**	%Tt.	RF	LF	RF	LF	RF	LF	RF	LF	RF	LF	RF	LF
Blister	12.8	0.0	4.0	8.0	7.7	22.2	27.8	0.0	5.3	5.3	0.0	6.7	13.3
Erosion	2.8	0.0	0.0	0.0	0.0	11.1	5.6	0.0	0.0	0.0	0.0	6.7	0.0
(0–14.5 km)		**Female**						**Male**					
		Tierra %		Set %		Cotton %		Tierra %		Set %		Cotton %	
**Muscle Lesion**	%Tt.	RF	LF	RF	LF	RF	LF	RF	LF	RF	LF	RF	LF
Pain due inadequate warm	21.1	23.1	27.8	8.0	7.7	27.8	27.8	10.5	15.8	10.5	0.0	13.3	13.3
Inflammation	2.8	0.0	4.2	4.0	0.0	0.0	0.0	0.0	10.5	10.5	0.0	0.0	0.0
Muscle discomfort	1.8	0.0	0.0	0.0	0.00	0.0	0.0	5.3	5.3	0.0	0.0	0.0	0.0

Km = Kilometers, % = Percentage, Tt = Total, RF = Right foot, LF = Left foot.

**Table 4:** Prevalence of skin, muscle, and nail lesions during the 14.5 to 29.6 km of the hike by sex.

(14.5–29.6 km)		**Female**						**Male**					
		Tierra %		Set %		Cotton %		Tierra %		Set %		Cotton %	
**Skin Lesion**	%Tt.	RF	LF	RF	LF	RF	LF	RF	LF	RF	LF	RF	LF
Blister	19.6	7.7	12.0	20.0	7.7	25.0	12.5	5.3	0.0	5.3	21.1	6.7	0.0
Erosion	7.5	0.0	4.0	8.0	0.0	12.5	18.8	0.0	0.0	0.0	0.0	20.0	13.3
Reddened skin	3.7	0.0	0.0	0.0	0.0	18.8	18.8	0.0	0.0	0.0	0.0	6.7	6.7
Urticaria	0.9	0.0	0.0	0.0	0.0	6.3	6.3	0.0	0.0	0.0	0.0	0.0	0.0
Crevice	0.9	0.0	0.0	0.0	0.0	0.0	0.0	0.0	0.0	0.0	0.0	6.7	0.0
Heloma	0.9	0.0	0.0	0.0	0.0	0.0	0.0	0.0	0.0	0.0	0.0	6.7	6.7
(14.5–29.6 km)		**Female**						**Male**					
		Tierra %		Set %		Cotton %		Tierra %		Set %		Cotton %	
**Muscle Lesion**	%Tt.	RF	LF	RF	LF	RF	LF	RF	LF	RF	LF	RF	LF
Pain due inadequate warm	11.2	15.4	4.0	4.0	7.7	0.0	12.5	10.5	0.0	5.3	10.5	0.0	0.0
Inflammation	0.9	0.0	4.0	4.0	0.0	0.0	0.0	0.0	0.0	0.0	0.0	0.0	0.0
Muscle discomfort	0.9	0.0	0.0	0.0	0.0	0.0	0.0	0.0	0.0	5.3	0.0	0.0	0.0
Sprain	0.9	0.0	0.0	4.0	0.0	0.0	0.0	0.0	0.0	0.0	0.0	0.0	0.0
(14.5–29.6 km)		**Female**						**Male**					
		Tierra %		Set %		Cotton %		Tierra %		Set %		Cotton %	
**Nail lesion**	%Tt.	RF	LF	RF	LF	RF	LF	RF	LF	RF	LF	RF	LF
Onychocryptosis	0.9	7.7	0.0	0.0	0.0	0.0	0.0	0.0	0.0	0.0	0.0	0.0	0.0
Subungual hematoma	1.9	0.0	0.0	0.0	0.0	6.3	6.3	0.0	0.0	0.0	0.0	6.7	6.7

Km = Kilometres, % = Percentage, Tt = Total, RF = Right foot, LF = Left foot.

**Table 5:** Women’s foot temperature by sock type.

Female									
Distance (km)	Sock			*p*-Value Sock			*p*-Value Distance		
	T	S	C	T-S	T-C	S-C	T	S	C
**Dorsal Temp (°C) RF**									
0	34.7	35.0	19.9	1.000	0.000 *	0.000 *	(1) = 0.002 *	(1) = 0.000 *	(1) = 0.001 *
14.5	36.2	36.3	31.8	1.000	0.013 *	0.000 *	(2) = 0.000 *	(2) = 0.000 *	(2) = 0.000 *
29.6	36.3	36.3	30.6	1.000	0.000 *	0.000 *	(3) = 1.000	(3) = 1.000	(3) = 0.867
**Dorsal Temp (°C) LF**									
0	35.0	35.0	19.9	1.000	0.000 *	0.000 *	(1) = 0.000 *	(1) = 0.005 *	(1) = 0.003 *
14.5	36.4	36.2	32.2	0.931	0.002 *	0.036 *	(2) = 0.000 *	(2) = 0.001 *	(2) = 0.000 *
29.6	36.3	36.3	30.8	1.000	0.000 *	0.000 *	(3) = 1.000	(3) = 1.000	(3) = 0.472
**Plantar Temp (°C) RF**									
0	34.8	34.8	20.5	1.000	0.000 *	0.000 *	(1) = 0.007 *	(1) = 0.000 *	(1) = 0.000 *
14.5	36.0	36.2	29.7	1.000	0.000 *	0.000 *	(2) = 0.001 *	(2) = 0.000 *	(2) = 0.000 *
29.6	36.2	36.2	30.6	1.000	0.000 *	0.000 *	(3) = 1.000	(3) = 1.000	(3) = 1.000
**Plantar Temp (°C) LF**									
0	34.8	34.9	21.0	1.000	0.000 *	0.000 *	(1) = 0.002 *	(1) = 0.009 *	(1) = 0.000 *
14.5	36.2	36.0	30.2	0.287	0.000 *	0.000 *	(2) = 0.000 *	(2) = 0.000 *	(2) = 0.000 *
29.6	36.1	36.0	30.2	1.000	0.000 *	0.000 *	(3) = 0.420	(3) = 0.785	(3) = 1.000

Km = Kilometres, T = Tierra, S = Set, C = Cotton, Temp = Temperature, (°C) = Centigrade Degree, RF = Right foot, (*) = Significant Difference, LF = Left foot, (1) = Distance between 0 to 14.5 Km, (2) = Distance between 0 to 29.6, (3) = Distance between 14.5 to 29.6.

**Table 6:** Men’s foot temperature by sock type.

Male									
Distance (km)	Sock			*p*-Value Sock			*p*-Value Distance		
	T	S	C	T-S	T-C	S-C	T	S	C
**Dorsal Temp (°C) RF**									
0	34.9	35.1	22.0	1.000	0.000 *	0.000 *	(1) = 0.005 *	(1) = 0.017 *	(1) = 0.003 *
14.5	35.9	36.1	32.9	1.000	0.030 *	0.014 *	(2) = 0.000 *	(2) = 0.000 *	(2) = 0.000 *
29.6	36.3	36.4	30.0	1.000	0.000 *	0.000 *	(3) = 0.470	(3) = 0.223	(3) = 0.896
**Dorsal Temp (°C) LF**									
0	35.2	35.3	21.4	1.000	0.000 *	0.000 *	(1) = 0.008 *	(1) = 0.240	(1) = 0.003 *
14.5	36.3	36.0	32.5	0.931	0.036 *	0.002 *	(2) = 0.000 *	(2) = 0.003 *	(2) = 0.000 *
29.6	36.5	36.4	30.8	1.000	0.000 *	0.000 *	(3) = 0.128	(3) = 0.401	(3) = 0.558
**Plantar Temp (°C) RF**									
0	34.9	35.1	22.0	1.000	0.000 *	0.000 *	(1) = 0.011 *	(1) = 0.017 *	(1) = 0.004 *
14.5	35.8	36.4	30.3	0.249	0.007 *	0.000 *	(2) = 0.000 *	(2) = 0.000 *	(2) = 0.001 *
29.6	36.2	36.4	29.0	1.000	0.000 *	0.000 *	(3) = 0.091	(3) = 0.223	(3) = 1.000
**Plantar Temp (°C) LF**									
0	35.0	34.9	21.8	1.000	0.000 *	0.000 *	(1) = 0.006 *	(1) = 0.001 *	(1) = 0.042 *
14.5	36.1	35.7	31.2	0.287	0.000 *	0.000 *	(2) = 0.000 *	(2) = 0.000 *	(2) = 0.000 *
29.6	36.4	36.2	29.3	1.000	0.000 *	0.000 *	(3) = 0.056	(3) = 0.401	(3) = 0.558

Km = Kilometers, T = Tierra, S = Set, C = Cotton, Temp = Temperature, (°C) = Centigrade Degrees, RF = Right foot, (*) = Significant Difference, LF = Left Ffot, (1) = Distance between 0 to 14.5 Km, (2) = Distance between 0 to 29.6, (3) = Distance between 14.5 to 29.6.

**Table 7:** Probability of suffering skin and muscle lesions in relation to anthropometric variables and sock type throughout the hike.

Probability Skin Lesions			Probability Muscle Lesions	
Distance		Distance: 14.5–29.6 km	Distance: 0–14.5 km	
96% If female		90% If male	100% If male	
100% If non-technical sock		100% If non-technical sock	67% If non-technical sock	
Direct relation	Inverse relation	Inverse relation	Direct relation	Inverse relation
BMI	Age	Age	BMI	Age

km = Kilometer, % = Percentages, BMI = Body mass index.

## Supplementary Materials

The following are available online at https://www.mdpi.com/1660-4601/16/10/1871/s1. Protocol of Measures, Table S1: Foot temperature by shock type (technical/non-technical). Table S2: Foot temperature by shock composition.
