# Supplementary material for: Importance of Sock Type in the Development of Foot Lesions on Low-Difficulty, Short Hikes
Source: Int J Environ Res Public Health. 2019 May 27;16(10):1871. doi: 10.3390/ijerph16101871 (PMC6572579; doi:10.3390/ijerph16101871)
Supplement: Supplementary file 1 [file ijerph-16-01871-s001.pdf]

Supplementary data

Protocol of Measures. Top shows the protocol of measure at the starting point (0 km). Middle part includes the protocol of the first part of the hike (0-14.5 km) and lower part of the table reflex the measurement protocol of the second part of the hike (14.5-29.6 km)

PROTOCOL OF MEASURES

STARTING POINT (0 Km)

Dorsal Number:……………………………..Gender:…………………..Foot size:………………… Weight………Size………..
Body mass index (BMI):……………..
Nº Bag with Socks:…………………………. Previous Alterations on skin, muscles or nails...

Temperature RF **Temperature LF**

Plantar				Plantar			
Dorsal				Dorsal			

Perimeters RF: (Three different measures)

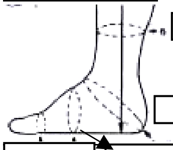

tibia
hell
toes
instep

Perimeters LF: (Three different measures)

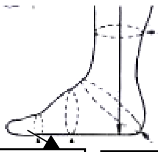

tibia
hell
toes
instep

FIRST PART OF THE HIKE (0-14.5 Km)

Temperature RF: (Three different measures)

Plantar			
Dorsal			

Temperature LF: (Three different measures)

Plantar			
Dorsal			

LESIONS (0-14.5KM)

SKIN LESIONS (SPECIFY RF AND LF)

Hyperkeratosis ……………… Heloma...............
Blister ………….Erosion……… Reeddned skin…………
Urticaria……..Grevice…………….. Others…………………

MUSCLE LESIONS (SPECIFY RF AND LF)

Pain due inadequate warm …………Inflamation..……
Muscle discomfort………… Sprain…… Others …..............

NAIL LESIONS (SPECIFY RF AND LF)

Onichocryptosis ………….subungual hematoma………
Others……………………………………………

Perimeters RF: (Three different measures)

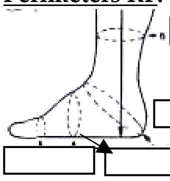

tibia
hell
toes
instep

Perimeters LF: (Three different measures)

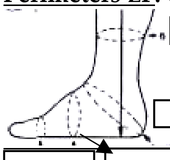

tibia
hell
toes
instep

EXTRA DETAILS

SECOND PART OF THE HIKE (14.5-29.6 Km)

Temperature RF: (Three different measures)

Plantar			
Dorsal			

Temperature LF: (Three different measures)

Plantar			
Dorsal			

LESIONS (14.5-29.6 Km)

SKIN LESIONS (SPECIFY RF AND LF)

Hyperkeratosis ……………… Heloma...............Blister …………Erosion………Reeddned skin………… Urticaria

Grevice……………..Others…………………………………..
………..

MUSCLE LESIONS (SPECIFY RF AND LF)

Pain due inadequate warm …………Inflamation..……
Muscle discomfort………… Sprain…… Others…………...

NAIL LESIONS (SPECIFY RF AND LF)

Onichocryptosis ………….subungual hematoma ……….
Others…………………………………………………………
…

Perimeters RF: (Three different measures)

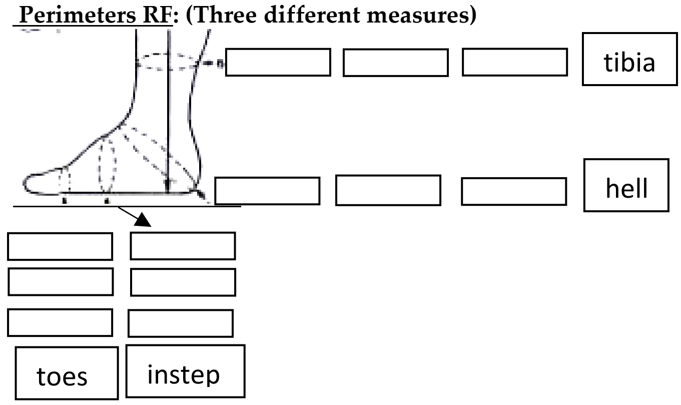

Perimeters LF: (Three different measures)

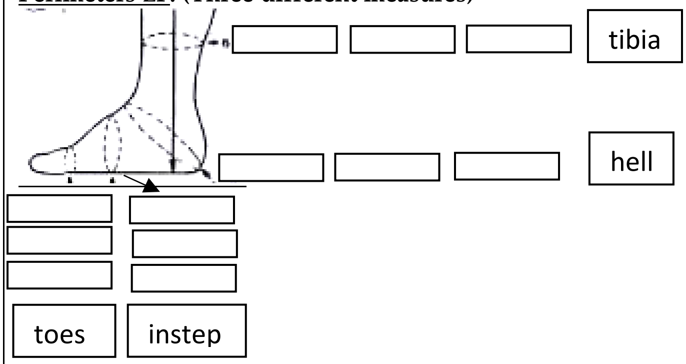

EXTRA DETAILS

Table S1. Foot temperature by sock type (technical/non-technical).

Distance	Socks		P-value socks	P-value distance	
**(km)**	**T**	**NT**	**T-NT**	**T**	**NT**
			**Dorsal temp (ºC) RF**		
**0**	35.0	20.7	0.000*	(1)=0.000*	(1)=0.000*
**14.5**	36.2	32.3	0.000*	(2)=0.000*	(2)=0.000*
**29.6**	36.3	30.4	0.000*	(3)=0.101	(3)=0.413
			**Dorsal temp (ºC) LF**		
**0**	35.2	20.6	0.000*	(1)=0.000*	(1)=0.000*
**14.5**	36.2	32.4	0.000*	(2)=0.000*	(2)=0.000*
**29.6**	36.4	30.8	0.000*	(3)=0.101	(3)=0.053
			**Plantar temp (ºC) RF**		
**0**	34.9	21.2	0.000*	(1)=0.000*	(1)=0.000*
**14.5**	36.0	30.0	0.000*	(2)=0.000*	(2)=0.000*
**29.6**	36.2	29.8	0.000*	(3)=0.091	(3)=0.847
			**Plantar temp (ºC) LF**		
**0**	34.9	21.3	0.000*	(1)=0.000*	(1)=0.000*
**14.5**	36.0	30.6	0.000*	(2)=0.000*	(2)=0.000*
**29.6**	36.2	29.8	0.000*	(3)=0.340	(3)=0.736

T=Technical, NT=Non-Technical, Km=Kilometres, Temp=Temperature, (ºC)=Degrees centigrade (*)=Significant difference, RF=Right foot, LF=Left foot, (1)= Distance between 0-14.5 Km, (2)=Distance between 0-29.6 Km, (3)= between 14.5-29.6 Km.

Table S2. Foot temperature by sock composition.

Distance (km)	Sock			P-value			P-value distance		
	T	S	C	T-S	T-C	S-C	T	S	C
					**Dorsal temp (ºC) RF**				
**0**	34.9	35.1	21.2	1.000	0.000*	0.000*	(1)=0.000*	(1)=0.000*	(1)=0.000*
**14.5**	36.1	36.2	32.3	1.000	0.000*	0.000*	(2)=0.000*	(2)=0.000*	(2)=0.000*
**29.6**	36.3	36.4	30.4	1.000	0.000*	0.000*	(3)=0.526	(3)=0.305	(3)=0.413
					**Dorsal temp (ºC) LF**				
**0**	35.1	35.2	20.6	1.000	0.000*	0.000*	(1)=0.000*	(1)=0.002*	(1)=0.000*
**14.5**	36.3	36.1	32.4	0.417	0.000*	0.001*	(2)=0.000*	(2)=0.00*	(2)=0.000*
**29.6**	36.4	36.4	30.8	1.000	0.000*	0.000*	(3)=0.341	(3)=0.467	(3)=0.158
					**Plantar temp (ºC) RF**				
**0**	34.9	35.0	21.2	1.000	0.000*	0.000*	(1)=0.000*	(1)=0.000*	(1)=0.000*
**14.5**	35.7	36.1	30.0	0.228	0.000*	0.000*	(2)=0.000*	(2)=0.000*	(2)=0.000*
**29.6**	36.2	36.3	29.9	1.000	0.000*	0.000*	(3)=0.826	(3)=0.826	(3)=1.000
					**Plantar temp (ºC) LF**				
**0**	34.9	34.9	21.3	1.000	0.000*	0.000*	(1)=0.000*	(1)=0.000*	(1)=0.000*
**14.5**	36.2	35.8	30.6	0.105	0.000*	0.000*	(2)=0.000*	(2)=0.000*	(2)=0.000*
**29.6**	36.2	36.1	29.8	1.000	0.000*	0.000*	(3)=1.000	(3)=0.184	(3)=0.736

Km=Kilometres, T=Tierra, S=Set, C=Cotton, temp=Temperature, (ºC)=Degrees centigrade, RF=Right foot, LF=Left foot, (*)=Significant difference, (1)= Distance between 0-14.5 Km, (2)= Distance between 0-29.6 Km, (3)= Distance between 14.5-29.6 Km.
